# Bumblebee electric charge stimulates floral volatile emissions in *Petunia integrifolia* but not in *Antirrhinum majus*

**DOI:** 10.1007/s00114-021-01740-2

**Published:** 2021-09-14

**Authors:** Clara Montgomery, Jozsef Vuts, Christine M. Woodcock, David M. Withall, Michael A. Birkett, John A. Pickett, Daniel Robert

**Affiliations:** 1 School of Biological Sciences, Life Sciences Building, University of Bristol, 24 Tyndall Avenue, Bristol, BS8 1TQ UK; 2 Department of Biointeractions and Crop Protection, Rothamsted Research, West Common, Harpenden, AL5 2JQ UK; 3 School of Chemistry, Cardiff University, Cardiff, CF10 3AT UK

**Keywords:** Plant,, Pollination,, Scent,, Signaling,, Triboelectricity

## Abstract

The timing of volatile organic compound (VOC) emission by flowering plants often coincides with pollinator foraging activity. Volatile emission is often considered to be paced by environmental variables, such as light intensity, and/or by circadian rhythmicity. The question arises as to what extent pollinators themselves provide information about their presence, in keeping with their long co-evolution with flowering plants. Bumblebees are electrically charged and provide electrical stimulation when visiting plants, as measured via the depolarisation of electric potential in the stem of flowers. Here we test the hypothesis that the electric charge of foraging bumblebees increases the floral volatile emissions of bee pollinated plants. We investigate the change in VOC emissions of two bee-pollinated plants (*Petunia integrifolia* and *Antirrhinum majus*) exposed to the electric charge typical of foraging bumblebees. *P. integrifolia* slightly increases its emissions of a behaviorally and physiologically active compound in response to visits by foraging bumblebees, presenting on average 121 pC of electric charge. We show that for *P. integrifolia,* strong electrical stimulation (600–700 pC) promotes increased volatile emissions, but this is not found when using weaker electrical charges more representative of flying pollinators (100 pC). Floral volatile emissions of *A. majus* were not affected by either strong (600–700 pC) or weak electric charges (100 pC). This study opens a new area of research whereby the electrical charge of flying insects may provide information to plants on the presence and phenology of their pollinators. As a form of electroreception, this sensory process would bear adaptive value, enabling plants to better ensure that their attractive chemical messages are released when a potential recipient is present.

**Supplementary Information:**

The online version contains supplementary material available at 10.1007/s00114-021-01740-2.

## Introduction

Olfaction is generally considered to be pivotal in underpinning plant-pollinator communication. Volatile organic compounds (VOCs) produced by flowering plants fulfil a large number of communicative roles (Dudareva et al. 2006; Das et al. 2013) are often highly species-specific (Pichersky and Gershenzon 2002) and can be indicative of pollination status (Theis and Raguso 2005). Diverse and ubiquitous, VOCs serve both intra- and inter-species communication (Karban et al. 2000; Dicke and Bruin 2001), advertising nectar and pollen availability and attracting pollinators across great distances (Haverkamp et al. 2016). In effect, many plant species are known to time their scent release with the foraging periods of their pollinators (Matile and Altenburger 1988; Dudareva et al. 2000; Hoballah et al. 2005; Theis et al. 2007; Bloch et al. 2017), thus presumably minimising unnecessary VOC synthesis (Raguso 2016). In some flowering plants, such as *Antirrhinum majus*, rhythmic scent emission persists in continuous light or dark conditions suggesting an endogenous rhythm independent of environmental influence (Kolosova et al. 2001). This is presumed to improve synchronicity between plants and pollinators (Bloch et al. 2017), yet sole reliance on an endogenous rhythm could allow VOC emissions when pollinators are absent, such as during rain or poor weather, where temporal or environmental cues stimulate volatile release (Helmig et al. 1998) but there is no reproductive benefit to the plant. Some diurnally flowering species, such as *Petunia integrifolia* and *Trifolium repens,* modulate their emissions of attractive scent based on environmental cues such as light intensity, which likely correspond to the abundance of some pollinators, but not all (Jakobsen and Olsen 1994; Hoballah et al. 2005). An efficient way to direct metabolic investment would be for flowers to sense the presence of their pollinators and gather fine temporal information to coordinate volatile emissions with pollinator activity. The process could have adaptive value as it would reduce unnecessary and wasteful volatile release whilst maximising chances of successful pollination (Raguso 2016). Reactive increases in volatile emissions in response to insect activity have been shown as a response to herbivory (Kessler and Baldwin 2001) but have not yet been investigated for pollination. Recently, evidence has surfaced that flowers respond to the vibrations produced by flying pollinators with an increase in nectar sweetness, providing the first evidence that flowers may sense and react to pollinator presence (Veits et al. 2019).

Altogether, foraging pollinators expose flowers to mechanical (Veits et al. 2019), chemical (Wetherwax 1986) and electrical stimulation (Clarke et al. 2013). Some pollinators, such as bees, are electrically charged in nature (Colin et al. 1991; Clarke et al. 2013; Montgomery et al. 2019). This charge attracts pollen and promotes its adhesion to the bee, facilitating the transportation of pollen between plants and enhancing pollination efficiency (Corbet et al. 1982; Armbruster 2001). In bumblebees, charge may also be constitutive to sensing weak electric fields via the deflection of mechanosensory hairs (Sutton et al. 2016). Bumblebees typically generate a positive electric charge, up to 1 nC in nature (Montgomery et al. 2019), but normally less than 100 pC in the laboratory (Clarke et al. 2013). A bee visiting a flower causes a depolarisation in the stem potential, which slowly declines after some time (Clarke et al. 2013). The visit of a charged bee to a flower may therefore provide specific information about the presence of pollinators via summation of these electric signals.

Here, we investigate the effect of electrical stimulation on the volatile emissions of two plant species: *Petunia integrifolia* (Hook.) Schinz & Thell. (Solanaceae) and *Antirrhinum majus* L. “Maryland True Pink” (MTP) cultivar (Plantaginaceae). We firstly test the hypothesis that the presence of foraging bumblebees increases the emission of attractive volatiles in *P. integrifolia*. We then test the hypothesis that electrical stimulation alone causes an increase in floral volatile emissions and test whether bumblebees can sense and respond to the VOCs produced during electrical stimulation. Finally, we test whether electrical stimulation causes an increase in volatile emissions in a plant with a more complex floral scent profile using *A. majus* MTP.

## Materials and methods

### Bee and flower maintenance

*Petunia integrifolia* and *Antirrhinum majus* MTP plants were grown from seed in the GroDome at the University of Bristol at a 16:8 day:night cycle at 20°C. Where experiments were conducted at Rothamsted, plants were transported from Bristol and housed in the Rothamsted greenhouses with a natural light cycle and kept at 22°C. Bumblebees (*Bombus terrestris audax* L.) were obtained from Koppert, UK, and were housed in the laboratory and trained to forage in a Perspex arena (100 × 75 × 40 cm) under a 16:8 day:night. Bees were provided with ad lib pollen (Bee Pollen Mixed Polifloral, The Happy Health Company, UK) and 30% sucrose solution.

### Dynamic headspace collection of floral volatiles

Volatiles were collected from both *P. integrifolia* and *A. majus* MTP by dynamic headspace collection (air entrainment), using Pye volatile collection kits (Kings Walden, Herts, UK). Intact flowers on potted and lightly watered *P. integrifolia* plants, and inflorescences of stem-cut *A. majus* plants placed in a conical flask containing water, were used throughout. To prepare headspace extracts for gas chromatography (GC) and GC-Mass Spectrometry (GC-MS) analyses, the flowers were individually enclosed in roasting bags (28cm × 30cm; Sainsbury's Supermarkets Ltd, UK), which were connected with a charcoal-cleaned air source, supplying an inflow of 600 mL/min. The air was then drawn through a Porapak Q trap, consisting of 50 mg 50/80 mesh polymer (Supelco, Bellefonte, PA) sandwiched between glass wool plugs in a 24-mm inner diameter glass tube, at 500 mL/min at the air outlet for 2 h, with the Porapak Q tube placed at the floral opening 5 mm from petals. A room control was done without flowers present to identify peaks relating to potential contaminants. Only peaks that were reliably present in the floral samples, but not in the room control, were analysed and identified. Prior to use, roasting bags were baked for 2 h at 140°C, and Porapak Q tubes were conditioned by washing each with 4 mL diethyl ether and heating at 132°C under a stream of nitrogen. The volatiles were eluted from the polymer tubes by flushing them with 750 μL redistilled diethyl ether. The samples were then concentrated to 50 μL and stored at -20°C until analysis.

For experiments requiring electrical stimulation, the flower needed to be accessed by an electrical stimulus, so encapsulation inside an inert container was impractical. As such, the Porapak Q tube was placed very close (<2 mm) to the flower of interest, but the flower or inflorescence was not enclosed. Air was subsequently drawn through the polymer at 500 mL/min for 2 h. To control for environmental contamination, control samples from the room without the flowers present were taken and analysed before and after the experiment. The floral compounds previously identified from enclosed flowers were not present in the room controls (Fig. S1). Any compounds present in the room controls were not analysed in the floral samples.

### GC and GC-MS

For the identification of the compounds present in *P. integrifolia* and *A. majus* MTP, a Hewlett-Packard 5890 series II GC fitted with a capillary HP-1 GC column (50 m × 0.32 mm i.d., 0.52 μm film thickness; J&W Scientific, Folsom, CA) and equipped with a cool on-column injector was directly coupled to a mass spectrometer (Hewlett-Packard 5972 mass-selective detector). Ionisation was by electron impact at 70 eV, 220°C. The oven temperature was maintained at 40°C for 1 min and then programmed at 5°C/min to 250°C (hold time 17.2 min). The carrier gas was helium. Tentative identification by GC-MS was confirmed by comparing retention index of the unknown peak with that of synthetic compounds and by GC peak enhancement by co-injection with an authentic sample (Pickett 1990), using an Agilent 6890N GC equipped with a cool on-column injector, flame ionisation detector and a 50 m × 0.32 mm i.d., 0.52 μm film thickness HP-1 column. The oven temperature was maintained at 30°C for 1 min and then programmed at 5°C/min to 150°C for 0.1 min, then at 10°C/min to 250°C for 20 min. The carrier gas was hydrogen. Compounds were quantified using the single point external method with an *n*-alkane (C_7_-C_22_) mixture. Authentic standards were purchased from Sigma-Aldrich UK and were >95% pure according to the supplier`s guidelines. (*E*)-Ocimene was synthesized in our laboratory as previously described (Hassemer et al. 2016).

### Measuring the electric charges on bees and the change in VOC emission from *P. integrifolia*

**Fig. 1:**
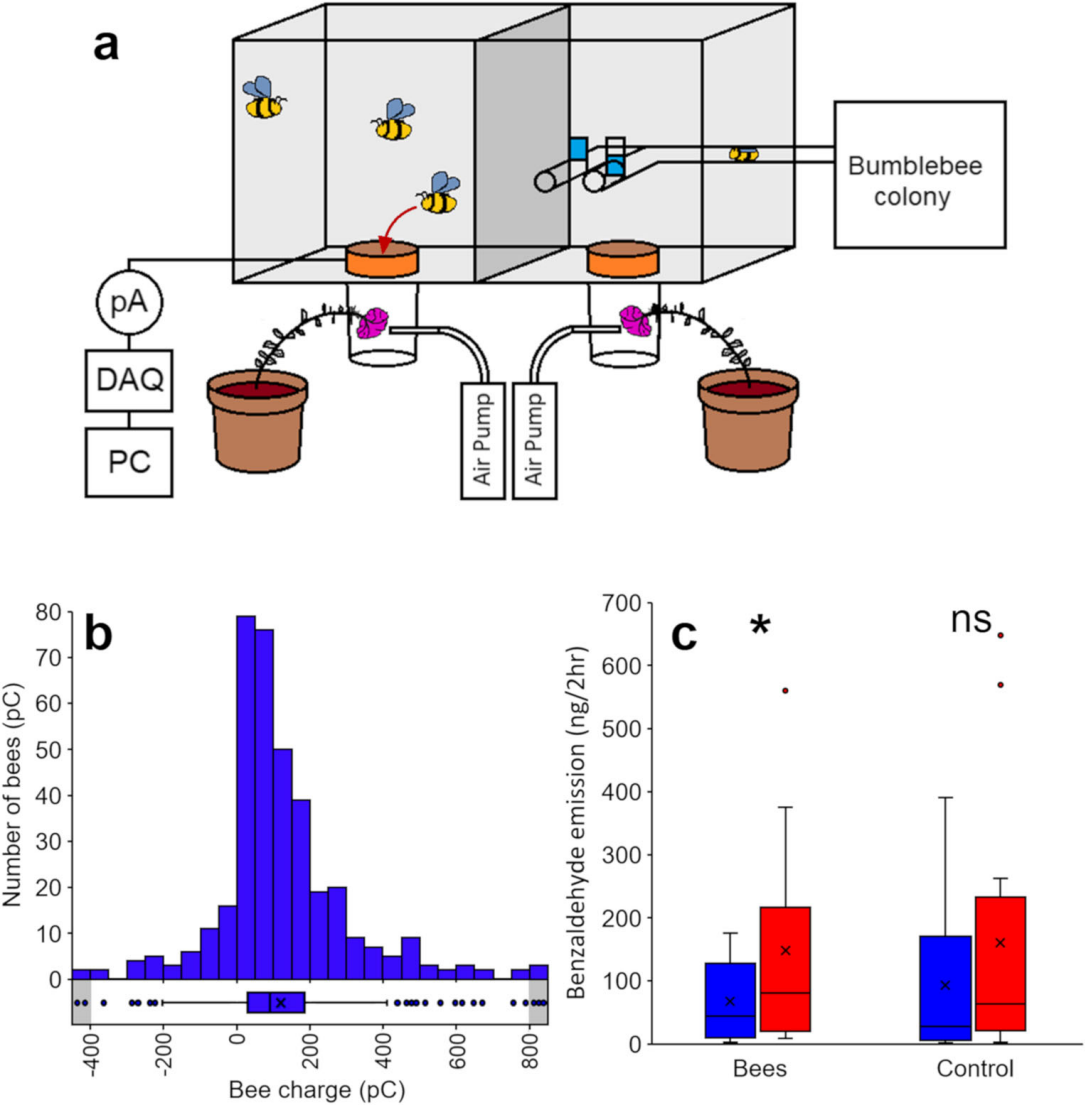
Testing *P. integrifolia* volatile emission in response to visitation by electrically charged pollinators (*Bombus terrestris*). **a** Experimental set up allowing bees to visit one *P. integrifolia* flower whilst the other acts as a control. The bee accesses the flower by flying through a metal ring in the floor of the arena. The charge on the bee induces a current in the ring, measured by a picoammeter (pA) connected to a computer via a data acquisition unit (DAQ). The volatiles are collected via air entrainment. **b** Distribution of electric charges of bumblebees approaching the *P. integrifolia* flowers throughout the experiment. Boxplot shows mean (X), median, SD, interquartile range, and outliers. Areas shown by grey zones encompass all values <-400 pC and >800 pC (range = 1041 pC to -832 pC, *N* = 377). **c** Quantitative measure of benzaldehyde emitted by the *P. integrifolia* flowers before (blue boxes) and during (red boxes) bee foraging, showing emissions of flowers visited by bees (left) and flowers touched with a grounded rod as a mechanical control (right), *N* = 12. Significance levels: ns not significant, * *P* < 0.05

*Bombus terrestris* bumblebees were trained to visit *P. integrifolia* flowers in a laboratory foraging arena. A bumblebee flight arena was split into two (Fig. 1a). Both sides were connected to a bumblebee colony via polyurethane tubes, which contained doors that could be closed and opened to control bee access to each side of the arena. Each side contained a ring charge sensor [RCS, described by Montgomery et al. 2019] consisting of an identical metal ring connected to a picoammeter. Each RCS was calibrated with a Faraday pail (JCI 141, Chilworth Global, Southampton, UK) to measure, in a non-contact manner, the charge on bees approaching the flower. Bees were initially trained to fly through each RCS to access a sugar reward.

During trials, a *P. integrifolia* flower was placed underneath each RCS, so that the bees would have to fly through the RCS to reach the flower (Fig. 1*A*). All bees were removed from the arena and volatiles were collected from both flowers for 2 h. The Porapak Q tubes were then refreshed and bees were allowed to forage in one side of the arena (and visit the experimental flower) but were excluded from the other side of the arena, so that only one flower could be visited by bees (Fig. 1*A*). Volatiles were collected from both flowers for a further 2 h. The charge on each bee visiting the experimental flower over the 2 h period was measured. Whenever a bee visited the experimental flower, the control flower was touched with a grounded rod to control for the mechanical stimulus. The increase in the amount of benzaldehyde produced by each flower was compared over the 2 h period before and after adding bees, using Wilcoxon signed rank tests for the experimental and control flowers. All statistical tests were conducted using R (version 3.5.1). One experimental and control flower was removed from analysis due to bees severing the flower 10 minutes after being released into the arena.

### Measuring bee charge using the RCS

The RCS comprised 2 concentric conductive aluminium rings based on the sensor described by Colin et al. (1991). These are insulated from each other by a layer of polycarbonate. The outer ring was electrically grounded and acted as an electrical shield, whilst the inner ring was connected to a picoammeter. When a charged object moved through the inner ring, it induced a current in the ring, the integral of which was proportional to the charge on the object passing through. Two RCSs were used to measure the charge on bees visiting *P. integrifolia* flowers. Each RCS was calibrated *in situ* by dropping charged polyurethane cubes (1 cm × 1 cm × 1 cm) through the RCS into a Faraday pail (JCI 141, Chilworth Global, Southampton, UK). The charge measured by each RCS and by the Faraday pail had a direct linear correlation with R^2^ values of 0.92 and 0.97.

### Manual electrical stimulation of flowers

To distinguish between the effects of electrical and mechanical stimulation, volatile emissions were measured from *P. integrifolia* flowers whilst either electrically stimulated by touching with a charged nylon ball, or mechanically stimulated by touching with an electrically grounded metal rod. Plants were randomly allocated to the control group (touched with electrically grounded rod) or the experimental group (electrically stimulated by touching with a positively charged rod). Plants with flowers of the same age were randomly paired into control and experimental groups. Flowers were used at 2–4 days post anthesis corresponding with the likely peak VOC emission period. All experiments took place between 9:00 and 17:00. To account for temporal variation, measurements were always taken from control and experimental plants simultaneously. During each trial a control and an experimental plant were placed at opposite ends of a room. Using a portable dynamic headspace sampling kit (Pye volatile collection kit, Kings Walden, Herts, UK), volatiles were collected from the control and experimental flowers for 2 h at a flow rate of 500 mLmin^-1^ by placing a Porapak Q tube at the opening of the flower 5 mm from the petals. The soil at the base of the plant was lightly watered before volatile collection took place. Volatiles were collected from both plants whilst undisturbed for 2 h. After this time, the soil was lightly watered again and the plants were electrically grounded by piercing the soil at the base of the plant with a grounded metal wire. The volatiles were collected for a further 2 h, during which the experimental flower was electrically stimulated every 10 min by lightly touching the flower with a positively charged ball. The stimulus carrier consisted of a nylon ball (diameter 10 mm) fixed to a wooden rod which was given an electric charge of approximately 1 nC by rubbing the ball with polystyrene. The charge on the ball was measured using a JCI 147 Faraday pail with a JCI 140 voltmeter (Chilworth Global, Southampton, UK) before and after touching the plant. The control flower was touched at the same 10 min intervals with a metal rod that was electrically grounded. The charge on the nylon ball dissipated rapidly. To estimate the charge on the ball at the point of contact with the flower, the charge decline on the ball was measured by charging the ball positively by triboelectrification and holding the ball in a Faraday pail (*n* = 5). An exponential decay curve was fitted to the data and used to estimate the charge on the ball at a point in time given the starting charge (Fig. S2). The increase in benzaldehyde produced by the flowers was compared using a Student’s paired *t*-test. With the low-charge experiment, the distribution of results was non-normal, so Wilcoxon-Mann-Whitney was used to compare the volatile emissions before and after stimulation.

For the electrical stimulation of *A. majus* MTP, 2 inflorescences were cut from each plant and placed in conical flasks containing water. A strip of aluminium foil connected to a grounding point was also placed in the water to electrically ground the base of the stem. Flowers of a similar age on each inflorescence were randomly allocated to be touched with the grounded rod or the experimental charged ball. The volatiles were then collected from the control and experimental inflorescences over a 2 h period, during which every 10 min, the outer lobe of the flower was touched with the grounded rod or charged ball. This experiment was done with separate inflorescences at both <1000 pC and <100 pC of charge. The rods were charged in an identical manner to the experiments with *P. integrifolia* and the charge was measured the same way. The amount of each volatile produced by the charged and the control flowers was compared. The amount of each volatile was highly correlated within each flower, so volatiles were combined for each flower and the total volatile emissions were compared.

### Behavioural responses of bumblebees to benzaldehyde

GC and GC-MS identified benzaldehyde as the primary compound produced by *P. integrifolia*. The ability of bumblebees to sense benzaldehyde was tested using the proboscis extension reflex (PER) and by coupled gas chromatography–electroantennography (GC-EAG). The PER experiment is a common behavioural experiment used to test memory and learning in insects. PER involves pairing a scent (conditioned stimulus) with a sugar reward (unconditioned stimulus). Over a series of trials, the bee is taught to associate the scent with the reward. During a trial, the bee is presented with the scent and given the opportunity to extend its proboscis (unconditioned response). The antenna of the bee is then touched with a tissue containing 30% sugar solution, causing the bee to extend its proboscis and the bee is allowed to drink from the sugar solution. Once the association is learnt, the bee will extend its proboscis in anticipation of the reward upon detecting the scent (conditioned response). An overview of PER in bumblebees is found in Laloi et al. (1999).

The PER experiment exposed bumblebees to the scent of benzaldehyde administered as a puff of air from a pipette containing a filter paper onto which 2 μL of pure benzaldehyde was applied. Bees were starved of sugar water 12 h prior to the experiment. One bee was anaesthetised using CO_2_ and placed in an enclosure formed from the head of a pipette, where the end had been removed to allow the head and tongue to protrude out the front of the enclosure. The bee enclosure and the end of the stimulus pipette were held down with plasticine modelling clay (TTS, UK). The stimulus pipette was placed so the tip was 1 cm away from the head of the enclosure. The reward was administered as a drop of 30% sugar water on cotton wool rolled around a wooden rod.

Sixteen bees were conditioned through 10 trials to associate the puff of air containing benzaldehyde with a reward (administered as a small drop of 30% sugar water on tissue paper wrapped around a wooden rod). Each trial consisted of slowly depressing the stimulus pipette for 12 seconds ensuring flow of scented air past the head of the bee. During the first 6 s of this period, the bee was observed for proboscis extension. During the second 6 s, the bee was presented with a sugar solution by lightly touching the antenna with the solution and allowed to drink.

The bee was left for 5 min between trials to allow the benzaldehyde scent to dissipate. After 10 conditioning trials, 3 control trials (Trial 11, 12 and 13) were administered, where the stimulus pipette was replaced by a control pipette not containing filter paper. In all but one case, these failed to elicit a PER response from the bee. After the 3 control trials, a final stimulus trial was conducted with the original benzaldehyde scent stimulus. The purpose of the control and final stimulus trials was to confirm the bee was responding to the scent of benzaldehyde and not just to the mechanical stimulus of the puff of air.

### Electrophysiological responses of bumblebees to floral volatiles

Volatiles were collected from enclosed *P. integrifolia* and *A. majus* MTP flowers by dynamic headspace collection (air entrainment). To locate the compounds that bumblebees responded to in headspace extracts from *P. integrifolia* and *A. majus* MTP, coupled GC-electroantennography (GC-EAG) was used. The system has been described previously (Wadhams 1990). EAG electrodes were constructed using borosilicate glass capillaries (2 mm outer diameter, 1.6 mm inner diameter) using a Narishige electrode puller (Optical Instrument Services Ltd, Croydon, UK). These were filled with electrolyte solution (7.55 gL^-1^ sodium chloride, 0.64 gL^-1^ potassium chloride, 0.22 gL^-1^ calcium chloride, 0.86 gL^-1^ sodium bicarbonate, 1.73 gL^-1^ magnesium chloride, 0.61 gL^-1^ sodium orthophosphate). The electrodes were attached to a holder (Ockenfels Syntech GmbH, Kirchzarten, Germany) on a micromanipulator (Leica Microsystems, Milton Keynes, UK) and threaded on so that a silver wire connected to the circuitry was inside the electrolyte.

A worker bumblebee was anaesthetised by cooling on ice, and an antenna was excised below the scape, also making a slit in the tip to ensure better contact between the electrolyte and the antenna. Either end of the excised antenna was placed in the tip of the electrodes. A glass tube with a hole in the side was placed 10 mm in front of the antenna, through which charcoal-filtered and humidified air was passed at a constant flow of 1 L/min. The effluent from the GC was split (1:1) between the flame ionisation detector (FID) and a heated GC transfer line (250°C) connected to the humidified air flowing towards the antennal preparation. The signals were passed through a high-impedance Syntech amplifier. Separation of VOCs collected from flower headspaces was achieved on a GC (6890N; Agilent Technologies, Santa Clara, CA) equipped with a cool-on-column injector and an FID, using a 50 m × 0.32 mm i.d. × 0.52 μm film thickness non-polar HP-1 column. The oven temperature was maintained at 30°C for 2 min and then programmed at 5°C/min to 250°C. The carrier gas was helium. The outputs from the EAG amplifier and the FID were monitored simultaneously and analysed using a customised software package (Syntech). One μL aliquots of pooled headspace samples were injected. A compound was identified as EAG-active if it evoked an antennal response in three coupled runs.

## Results

### Bee charge and volatile emissions

The bees visiting the flowers in the laboratory were predominantly positively charged (Fig. 1b; 87% positively charged, 13% negatively charged, *N* = 377, mean charge ± SE = 121 ± 9 pC).

Flowers visited by free-flying bumblebees exhibited a significant increase in volatile production (Paired Wilcoxon test, *P* = 0.021, *V* = 68, *N* = 12) (Fig. 1c). By contrast, flowers touched with an electrically grounded metal rod did not show such increase (Paired Wilcoxon test, *P* = 0.077, *V* = 6 2, *N* = 12) (Fig. 1c).

### Manual electrical stimulation and volatile emissions

In arena experiments, flowers visited by bumblebees underwent significant mechanical damage to their corolla (Fig. S4).

**Fig. 2:**
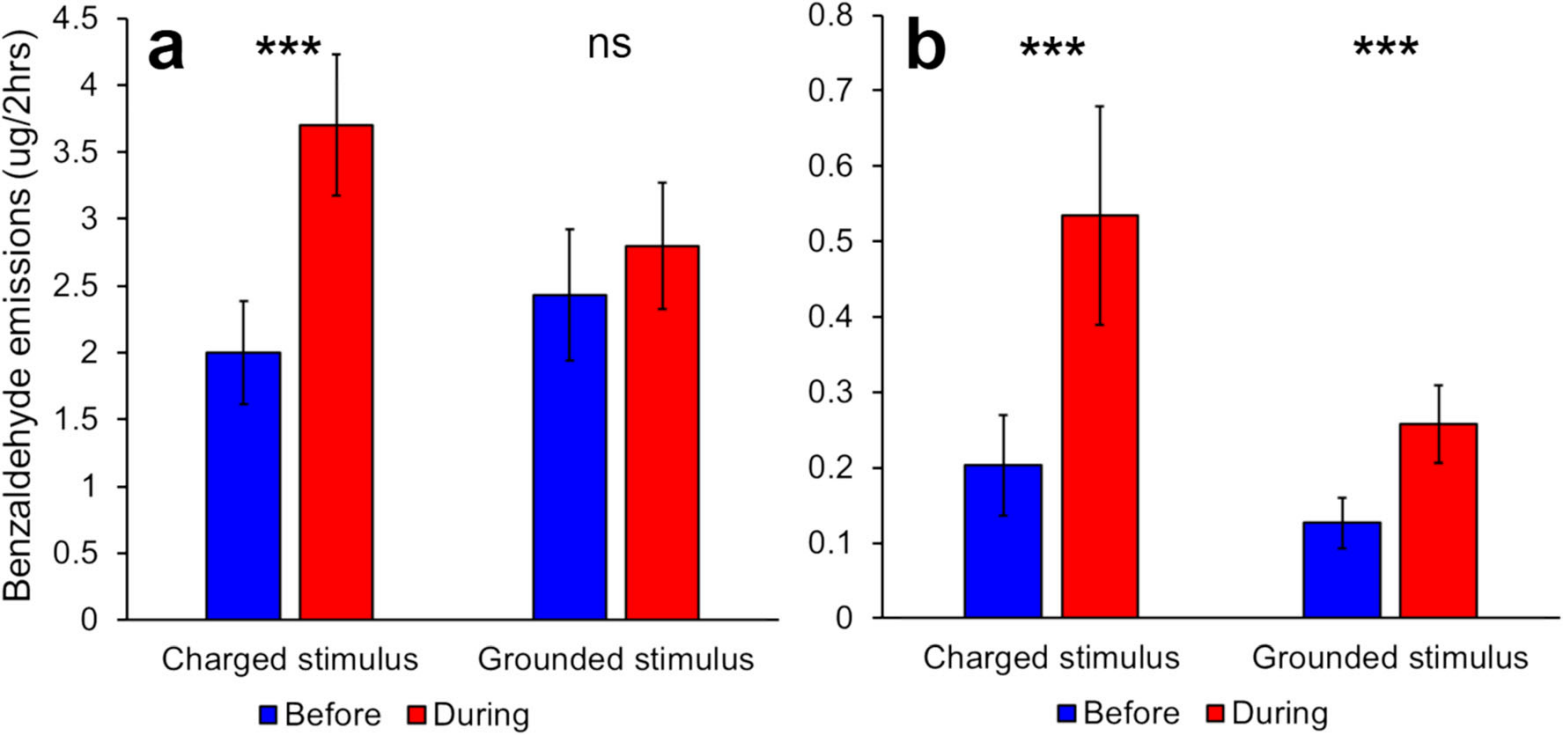
**a** Electrical stimulation with a triboelectrically charged nylon ball of 600–700 pC causes significant increase in benzaldehyde emissions from *P. integrifolia* flowers*,* whilst grounded rod does not (*N* = 15). **b** A nylon ball charged to <100 pC causes a significant increase in benzaldehyde emissions, but plants touched with the grounded control also showed a significant increase in emissions (*N* = 12). Significance levels: ns not significant, *** *P* < 0.001

The volatile emissions of *P. integrifolia* flowers was significantly increased when touched with a 600-700 pC ball (paired *t*-test; *P* < 0.0001, *t* = -5.701, *df* = 15) (Fig. 2a), whilst no increase was seen from flowers touched with the grounded control rod (paired *t*-test; *P* = 0.240, *t* = -1.223, *df* = 15). When plants were touched with a ball with a much lower charge (<100 pC) inside a Faraday cage, there was a significant increase in emissions from both the flowers touched with the charged ball (Paired Wilcoxon; *P* = 0.0005, *V* = 0, *N* = 12; Fig. 2a) and flowers touched with the grounded rod (Paired Wilcoxon; *P* = 0.001, *V* = 1, *N* = 12).

### Behavioural and electrophysiological responses of bumblebees to benzaldehyde

**Fig. 3:**
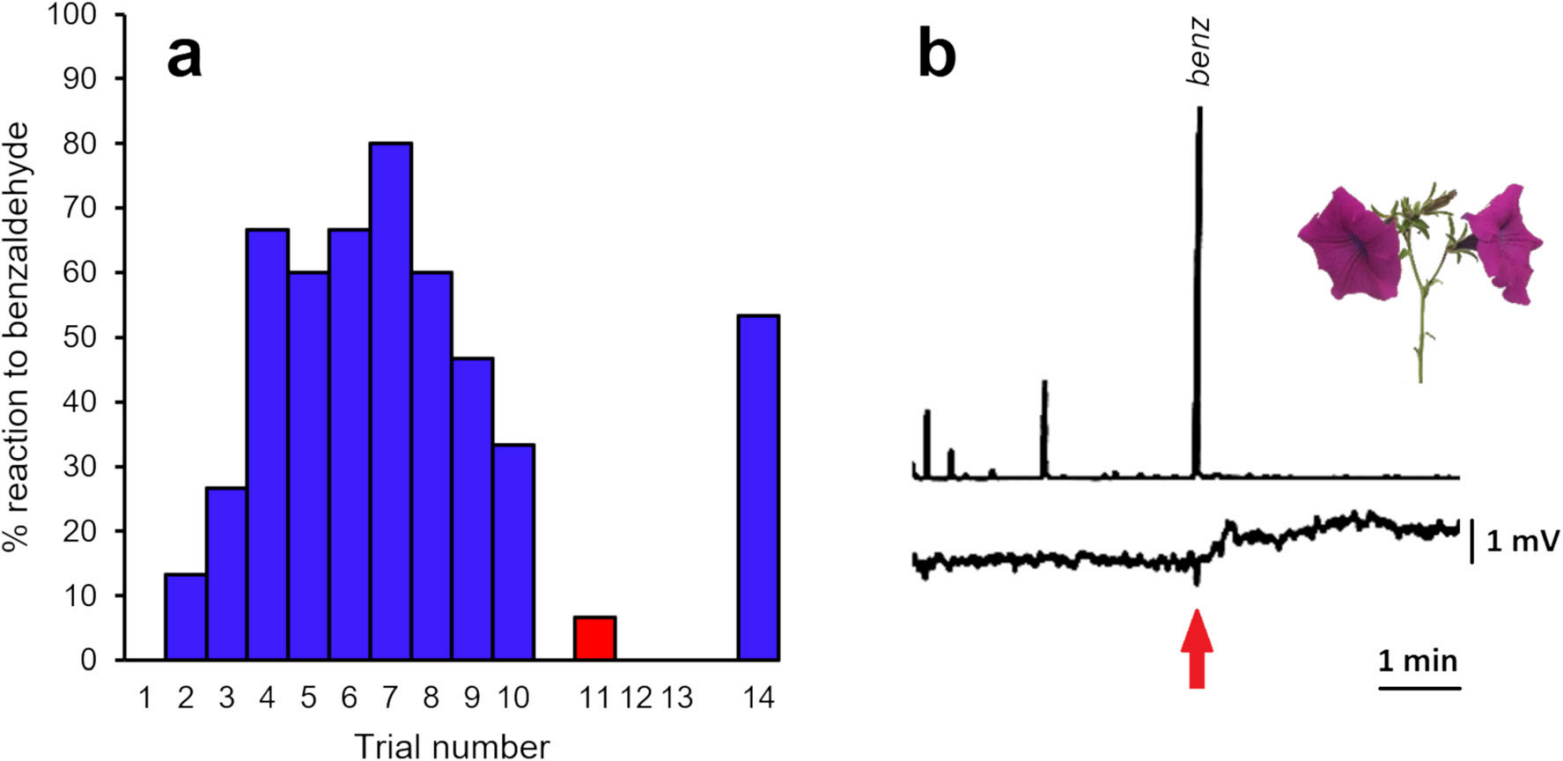
Behavioural and electrophysiological response of bumblebees to benzaldehyde. **a** PER responses of bumblebees to benzaldehyde. Trials 1-10 are training trials associating benzaldehyde scent with a sucrose reward. Trials 11-13 are control trials using unscented air. Trial 14 is a final confirmation trial. Data from 15 animals. **b** GC-EAG response of bumblebee antenna to benzaldehyde [Kováts retention index (KI) on a non-polar HP-1 GC column=943] present in a volatile sample taken from a *P. integrifolia* flower. Top trace represents GC/FID output with the large peak showing benzaldehyde. Red arrow on bottom trace indicates EAG response from a bumblebee antenna to the benzaldehyde peak

The repeated co-presentation of sucrose with benzaldehyde generated an associative conditioned response, behaviourally expressed as PER. The rate of PER response increased up to 80% following 7 trial presentations (Fig. 3a, *N* = 16) then declined to 38% after 10 trials. Unscented control trials failed to elicit a response in all but one case (1/16). The responses of bees over trials showed a gaussian distribution (Fig. 3a) suggesting possible fatigue, though the final scented trial had a 53% response rate, showing that the bees can reliably sense and respond to benzaldehyde.

Coupled GC-electroantennography (GC-EAG) was used to confirm that bumblebees could detect benzaldehyde, collected from *P. integrifolia,* by the peripheral olfactory system. Bumblebee antennae show distinct electrophysiological activity in response to benzaldehyde from *P. integrifolia* (Fig. 3b, *N* = 3).

### The response of Antirrhinum majus MTP to electrical stimulation

**Fig. 4:**
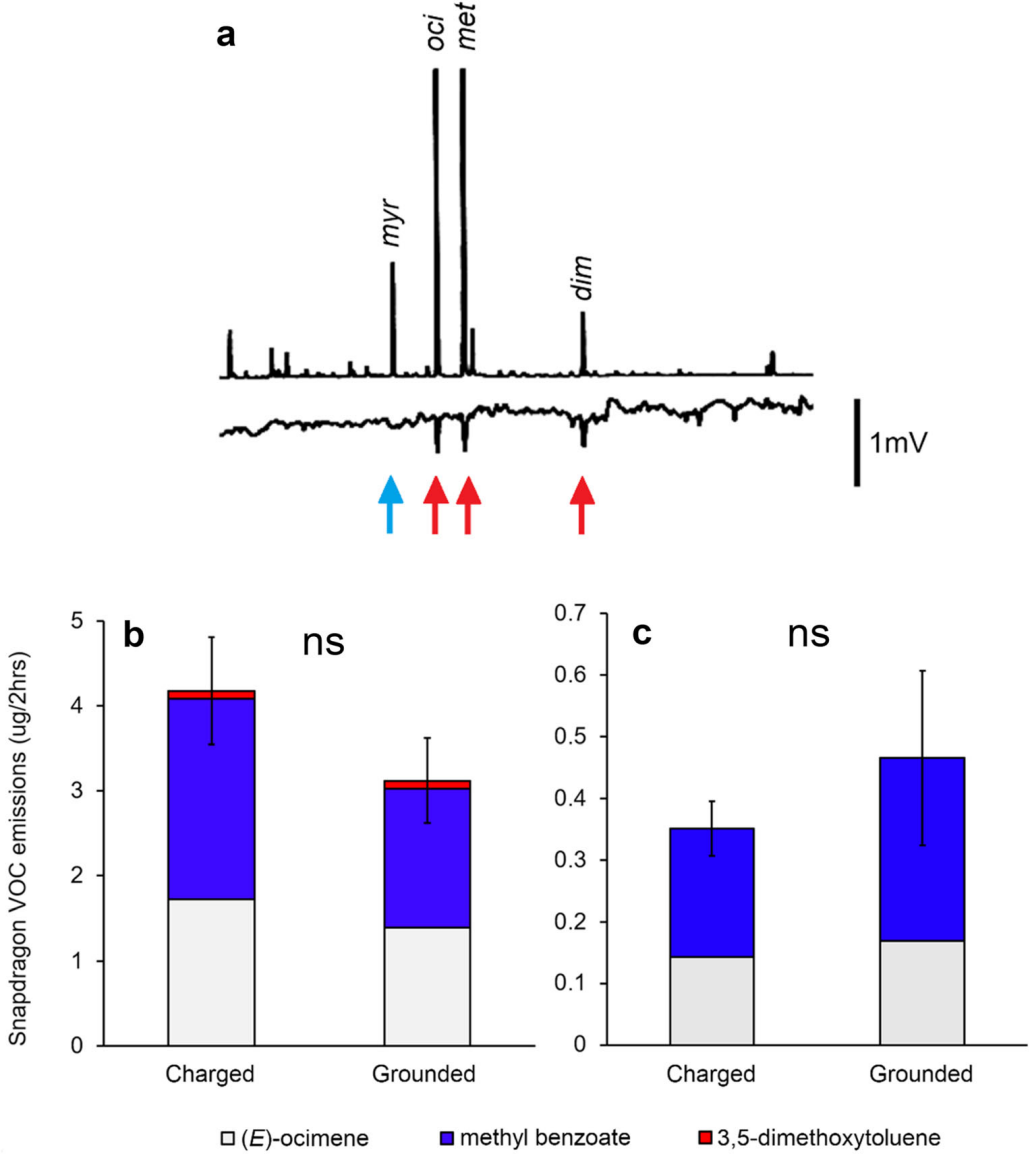
**a** The GC-EAG response of a bumblebee antenna to compounds present in *A. majus* MTP flower headspace extracts, showing FID peaks for myrcene (*myr,* KI=990), (*E*)-ocimene (*oci,* KI=1043), methyl benzoate (*met,* KI=1064) and 3,5-dimethoxytoluene (*dim,* KI=1246). Bottom trace shows EAG responses of a bumblebee antenna to (*E*)-ocimene, methyl benzoate and 3,5-dimethoxytoluene (red arrows), but no reaction is found for myrcene (blue arrow). **b** and **c** EAG-active floral volatiles produced by *A. majus* MTP when touched with a charged or grounded stimulus (*N* = 14). The charged stimulus was a nylon ball charged to 600-700 pC (**b**) and <100 pC (**c**). Significance levels: ns not significant

The capture of scents produced by *A. majus* MTP revealed 4 main compounds: myrcene, (*E*)-ocimene, methyl benzoate and 3,5-dimethoxytoluene (Fig. S3). These volatiles were first identified by both GC-MS and by their Kováts Indices and the identification was confirmed by GC peak enhancement on co-injection with authentic standards. Using the GC-EAG method, bumblebees were shown to respond to (*E*)-ocimene, methyl benzoate and 3, 5-dimethoxytoluene from *A. majus* MTP, but not to myrcene present in the same sample (Fig. 4a, *n*=3). *A. majus* MTP flowers touched with a charged ball did not emit greater quantities of volatiles than those touched with a grounded rod (High charge: paired *t*-test, *P* = 0.0935, *N* = 11, *t* = 1.854; Low charge: Wilcoxon, *P* = 0.8311, *N* = 11, *V* = 30). There was no difference in the ratio and diversity of compounds emitted from both stimulated and unstimulated plants.

## Discussion

The volatiles found to be produced by *P. intergrifolia* and *A. majus* MTP are consistent with those identified from these plants in previous studies (Dudareva et al. 2003; Hoballah et al. 2005), with benzaldehyde being the main compound produced by *P. integrifolia* (Fig. 1b; Hoballah et al. 2005). The behavioural and electrophysiological experiments collectively show that bumblebees can detect and behaviourally respond to the scent of benzaldehyde, which corroborates the generally accepted capacity of Apidae (Hymenoptera, including *Bombus* spp.) to be attracted to volatile blends containing benzaldehyde (Roy and Raguso 1997; El-Sayed et al. 2018; Ramos and Schiestl 2019). The three main compounds present in *A. majus* MTP were myrcene, (*E*)-ocimene and methyl benzoate (Fig. 4b), which is consistent with the compounds identified from this cultivar in the literature (Dudareva et al. 2000; Dudareva et al. 2003; Wright et al. 2005). For the first time, however, we find that bumblebees also show consistent electrophysiological responses to a fourth compound present in this cultivar, 3, 5-dimethoxytoluene (Fig. 4b), suggesting that this compound may play a previously overlooked role in the attraction of pollinators to *A. majus*.

The results presented here show for the first time that repeated visits by *B. terrestris* augment the emission of pollinator-attractive volatiles in *P. integrifolia* in a laboratory environment. Many plants modify their volatile emissions in response to biotic stresses such as predation (Kessler and Baldwin 2001), as well as environmental factors such as light and temperature (Cheng et al. 2016), but we show for the first time here that plants may use cues provided by their pollinators to modulate their emissions of attractive scent. For plants, real-time detection of pollinator presence would allow more effective targeting of volatile release rather than relying on environmental or temporal cues, which may not accurately reflect pollinator presence and abundance such as when volatiles are released in poor weather (Helmig et al. 1998). Direct sensing of pollinators would maximise reproductive success by ensuring maximum pollen dispersal whilst also minimising wasteful emissions when pollinators are not present. There is theorised a metabolic cost to producing VOCs (Hoballah et al. 2004), although metabolic cost is often dwarfed by the much higher cost of increased risk of detection by folivores and herbivores (Raguso 2016). Therefore, in addition to increasing pollinator attraction and achieving greater pollen dispersal, direct detection of pollinators may reduce the risk of attracting folivores and herbivores by benzaldehyde (Theis 2006; Theis et al. 2007). In effect, the direct detection of pollinators, using electric charge sensitivity or other cues such as pollinator-specific vibrations (Veits et al. 2019), could offer more reliable prediction of pollinator phenology than more correlational parameters such as temperature or luminosity, which are strongly affected by weather. Exclusion experiments conducted in a field setting would be instrumental in elucidating the sensory capabilities of flowering plants and the overlapping roles of electrical, mechanical, and chemical signalling in the plant-pollinator relationship.

Electrical stimulation with a strong electric charge causes an increase in benzaldehyde emission in *P. integrifolia*, suggesting that a strongly charged pollinator may induce greater volatile emissions in receptive plants. As pollinating insects have been consistently shown to carry a positive electric charge (Corbet et al. 1982; Colin et al. 1991), this increase in emissions would provide reproductive benefits to the plant by enhancing pollinator attraction and hence pollen dispersal, maximising the chances of successful cross-pollination. This charge-mediated increase in emissions could create a positive feedback loop, where visits from charged pollinators cause flowers to release more scent, attracting further pollinators. This would continue until the flowers’ nectar and pollen resources were depleted and all available pollen had been dispersed. Attracting strongly charged pollinators has an additional reproductive benefit to the plant: charged pollinators create an electric field between plant and pollinator, which encourages the bidirectional transfer of pollen through the air due to Coulomb force (Clarke et al. 2017). The shape of the floral electric field attracts this pollen directly to the stigma, maximising reproductive success (Clarke et al. 2017). Thus, a positive feedback loop attracting further charged pollinators to the flower would increase the rate of pollen dispersal, and increase the likelihood of efficient pollen transfer between plant and pollinator.

The electric charges measured on bumblebees approaching a petunia flower in the laboratory were similar in magnitude and distribution to those measured from outdoor free-flying bumblebees (Montgomery et al. 2019). Thus, arena-based foraging bumblebees presented a charge commensurate with that of bees foraging outdoors. Whilst pollinator charge is consistently positive, little is known about the charges on other insects (Clarke et al. 2017). Electric charge holds adaptive value for pollinators by increasing pollen attraction and adhesion (Corbet et al. 1982) and allowing sensing of electrostatic cues (Sutton et al. 2016). As flight has been shown to contribute to charge generation in insects (Edwards 1960; Erickson 1975), flying pollinators may have a greater electric charge than less aerial and agile herbivores. We therefore propose here that, as pollinators are found to be consistently electrically charged (Corbet et al. 1982; Colin et al. 1991; Montgomery et al. 2019), the detection and use of charge as an indicator of pollinator abundance has adaptive value for entomophilous plants. Frequent visitation of charged pollinators to a flower would cause charge summation perhaps exceeding a threshold for volatile release. Herbivorous insects, including folivores, meanwhile may not generate sufficient charges to exceed this threshold. Charge could therefore provide a useful indicator of pollinator abundance, allowing the plant to assess the real-time potential for pollen dispersal. Current understanding of the electric charges carried by different insect species is very low (Clarke et al. 2017). The electric charges carried by florivores feeding on *P. integrifolia,* such as cucumber beetles (*Diabrotica undecimpunctata*, Chrysomelidae) and tree crickets (*Oecanthus fultoni*, Gryllidae) (Kessler et al. 2013), would provide a useful comparison. Electric charges have been previously measured qualitatively on several insects including diptera, hymenoptera, lepidoptera and some coleoptera (Edwards 1962), but in highly artificial conditions with little consideration to how an electric charge may affect a species’ ecological role. A quantitative study comparing the electric charges on pollinators and herbivores would have great value in informing the different potential sensory cues that could allow plants to discriminate between beneficial and antagonistic insects.

The release of attractive floral volatiles changes over the lifetime of a flower. Post-pollination, plant volatiles sometimes decrease as the flower senesces and wilts. However, this takes place over many hours, sometimes days after pollination (Underwood et al. 2005). On a plant with multiple flowers, a short-term increase in volatile release could attract local pollinators and hence may cause increased pollinator visits to other unpollinated flowers on the same plant, enhancing the overall reproductive success. It is also possible that electric cues affect other floral modalities, such as nectar sweetness (Veits et al. 2019). Though the *P. integrifolia* flowers visited by bumblebees showed a significant increase in volatile emissions (Fig. 1c), the plants touched equivalently with a grounded metal rod also showed an increase in emissions approaching the arbitrary significance threshold (*P* = 0.077). This may indicate that the increase in benzaldehyde may be a stress response to the mechanical wear inflicted by bumblebees (Fig. S4). Whilst *P. integrifolia* is often used in bumblebee experiments, it is naturally pollinated by much smaller solitary bees (Ando et al. 2001). The increases in benzaldehyde emissions from electrically and mechanically stimulated *P. integrifolia* flowers may reflect their relative fragility and responsiveness to environmental stimuli. This explanation is supported by the lack of response seen with the more robust bumblebee-pollinated *A. majus,* the flowers of which can withstand significant damage from manipulation by bumblebees.

Variation in volatile emissions from individual *P. integrifolia* flowers under identical conditions can be substantial. For instance, daily volatile emissions of some individual flowers can be twice those of others under identical conditions (Negre et al. 2003), whilst the mean emissions of individual flowers have been shown to vary even under constant conditions (mean emissions 100-350 ng/4 h; Hoballah et al. 2005). To minimize this effect of individual variation in emissions, we compared each flower to itself with and without stimulation, allowing the addition of bees or mechanical stimulation to be the only affecting variable. The presence of outliers in the results therefore likely reflects the natural variation in emissions between flowers. Flowers were visually monitored throughout the experiment and data was only removed from analysis if it was justified by the scientific method. One result was removed from the live-bee experiment analysis as the bees severed the flower 10 minutes into the experiment. For all other instances the flowers were intact, so the data was all included for analysis, as there was no scientific basis for removal.

To analyse the effect of a weak electric charge on *P. integrifolia* volatile emissions, a low-charge experiment was conducted inside a Faraday cage to minimise external electrical interference. The Faraday cage dimensions necessitated that the plants were in close proximity (<1 m), potentially allowing some of the volatiles from the experimental plant to be collected by the apparatus near the control plant, as both plants were unenclosed. This may be responsible for the apparent increase in volatile emissions in the control *P. integrifolia* plants (Fig. 2b). This explanation is supported by the observation that the plants that were touched in the “High charge” experiment (including the control plants of both *P. integifolia* and *A. majus* MTP) had volatile emissions ten times greater than the equivalent plants in the “weak charge” experiment (Figs. 2, 4). Additionally, it is possible that the light intensity in the laboratory was higher than that in the greenhouse, and that the plants increased their emissions as a delayed response to the increased light, though this does not account for the differences in the *A. majus* MTP emissions. Finally, it can be pointed out that whilst the metallic rod was grounded using a grounding circuit independent of that of the main supply, residual charge present on the experimenter could have influenced both experimental and control plants.

*Antirrhinum majus* MTP flowers touched with an electric ball did not have greater volatile emissions than those touched with a grounded rod (Fig. 4). The morphology of *A. majus* MTP inflorescences necessitated a different experimental approach to the experiments done with *P. integrifolia*, due to the inability to isolate individual flowers. As such, different inflorescences of the same age were cut and compared whilst one was electrically stimulated and the other mechanically stimulated. This difference in approach (cut *A. majus* MTP plants vs potted *P. integrifolia*) may have affected the stem potential in the flowers, where electric charges were potentially conducted more rapidly away through the *A. majus* MTP plants. This was mitigated to the greatest extent by ensuring both cut and potted plants were as thoroughly grounded as possible. Cut flowers had an aluminium electrode placed in the water in the vase connected to ground. Potted plants were housed in damp soil with a grounded aluminium electrode placed in the soil 1 cm from the plant stem. The differences in response of *P. integrifolia* and *A. majus* MTP flowers may reflect differences in respective mechanisms of volatile synthesis and release. Electrical stimulation has been shown to increase plant VOC synthesis (Inaba et al. 1995; reviewed in Volkov 2017), but as plant volatiles must cross multiple membranes before release (Widhalm et al. 2015), changing membrane permeability could also cause greater volatile release. Adebesin et al. (2017) present an active transport mechanism in *Petunia hybrida,* where volatile compounds are transported across the plasma membrane via an adenosine triphosphate–binding cassette (ABC) transporter, PhABCG1 (Adebesin et al. 2017). Electric charging of floral tissues may therefore increase the activity of the ABC transporter, leading to increased benzaldehyde emissions in *P. integrifolia*.

The electric environment is ubiquitous and affects biological systems, from pollination and ecology to soil microbiota (Hunting et al. 2020), but the influence of electric fields on biological systems is often poorly understood and hard to quantify. These experiments indicate the need for future studies into the widespread effects of electric fields on flowering plants. Altogether, our results demonstrate the potential for the existence of a novel form of plant-pollinator interactions. The evolutionary significance of such a relationship, based on the plant’s ability to detect and integrate information carried by the electrical charge of visiting pollinators, is yet to be demonstrated. This discovery adds a new dimension to the rich catalogue of ways flowers interact with their pollinators (Jermy 1999, Gervasi and Schiestl 2017), and enhances our mechanistic understanding of plant-insect co-evolution.

## Supplementary information

Figure S1 The volatiles collected simultaneously over a 2 h period from *P. integrifolia* from **a** a flower touched with an electrically charged rod, **b** a flower touched with an electrically grounded rod and **c** the air in the room 1 m away from the flowers. Benzaldehyde peak indicated with red arrow. (JPEG 145 kb)

Figure S2 **a** The mean charge decline on a triboelectrically charged nylon ball held in a Faraday pail (blue), dashed lines show SD. Red line indicates the modelled relationship used to calculate the charge on the ball at the point of touching the flower. **b** The modelled charges present on the nylon ball at the point of touching the flowers during the high charge experiments. (TIFF 292 kb) (PNG 30926 kb)

High Resolution image (PNG 30926 kb) (TIFF 292 kb)

Figure S3 The major compounds present in **a** *P. integrifolia* and **b** *A. majus* MTP. Peak labels indicate benzaldehyde (*benz,* KI=946), myrcene (*myr,* KI=990), (*E*)-ocimene (*oci,* KI=1043), methyl benzoate (*met,* KI=1064) and 3,5-dimethoxytoluene (*dim,* KI=1246). (JPEG 2654 kb)

Figure S4 The same *Petunia integrifolia* flower before (**a**) and after (**b**) a 2 h exposure to bumblebees showing mechanical wear and damage from bumblebee tarsi. (JPEG 1976 kb)

## Data Availability

Data will be made available upon request.
