# Supplementary figures and images for: Bumblebee electric charge stimulates floral volatile emissions in Petunia integrifolia but not in Antirrhinum majus
Source: Naturwissenschaften. 2021 Sep 14;108(5):44. doi: 10.1007/s00114-021-01740-2 (PMC8440258; doi:10.1007/s00114-021-01740-2)

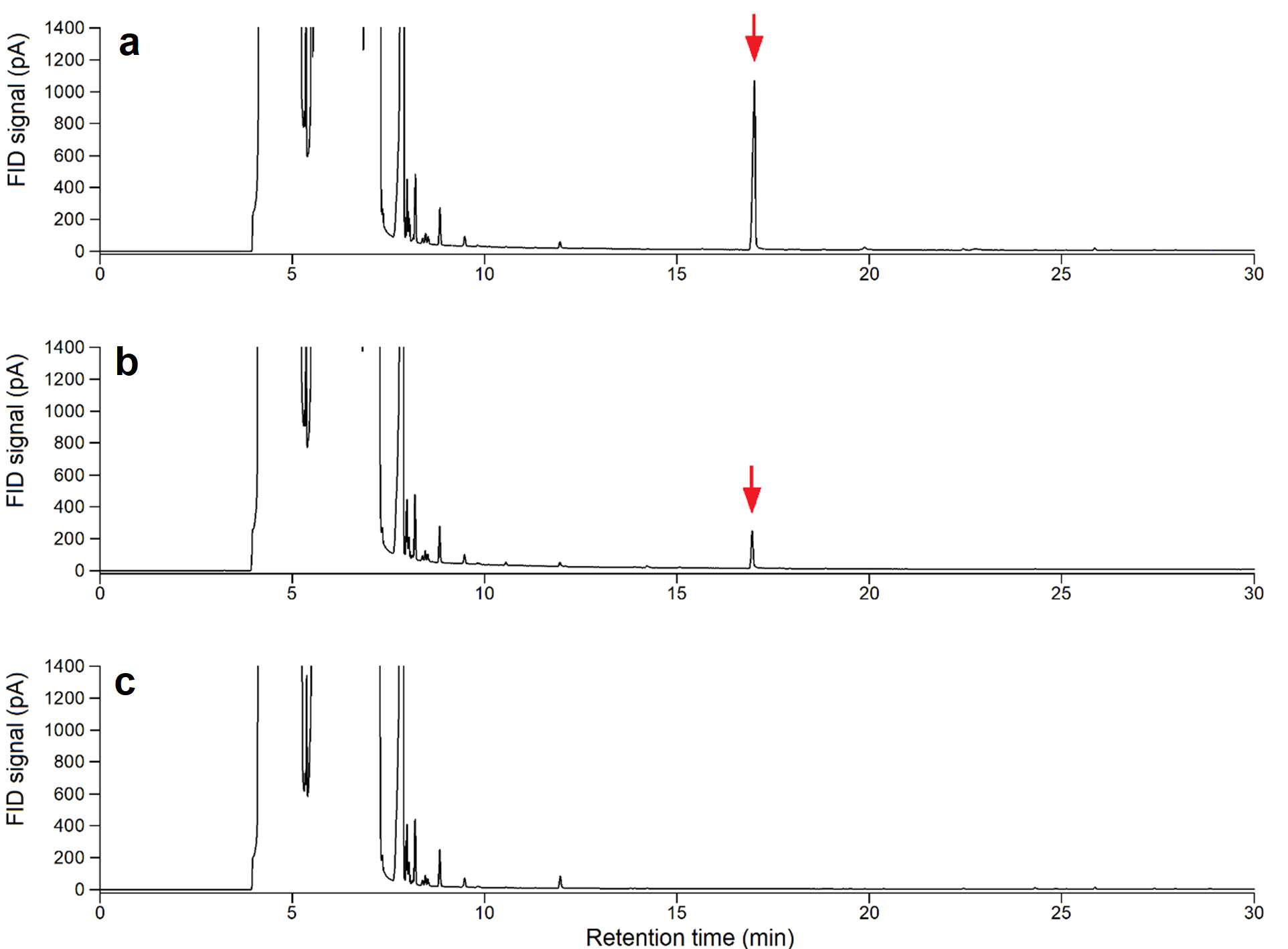

Supplement: Supplementary file 1 — The volatiles collected simultaneously over a 2 h period from P. integrifolia from a a flower touched with an electrically charged rod, b a flower touched with an electrically grounded rod and c the air in the room 1 m away from the flowers. Benzaldehyde peak indicated with red arrow. (JPEG 145 kb) [file 114_2021_1740_MOESM1_ESM.jpeg]

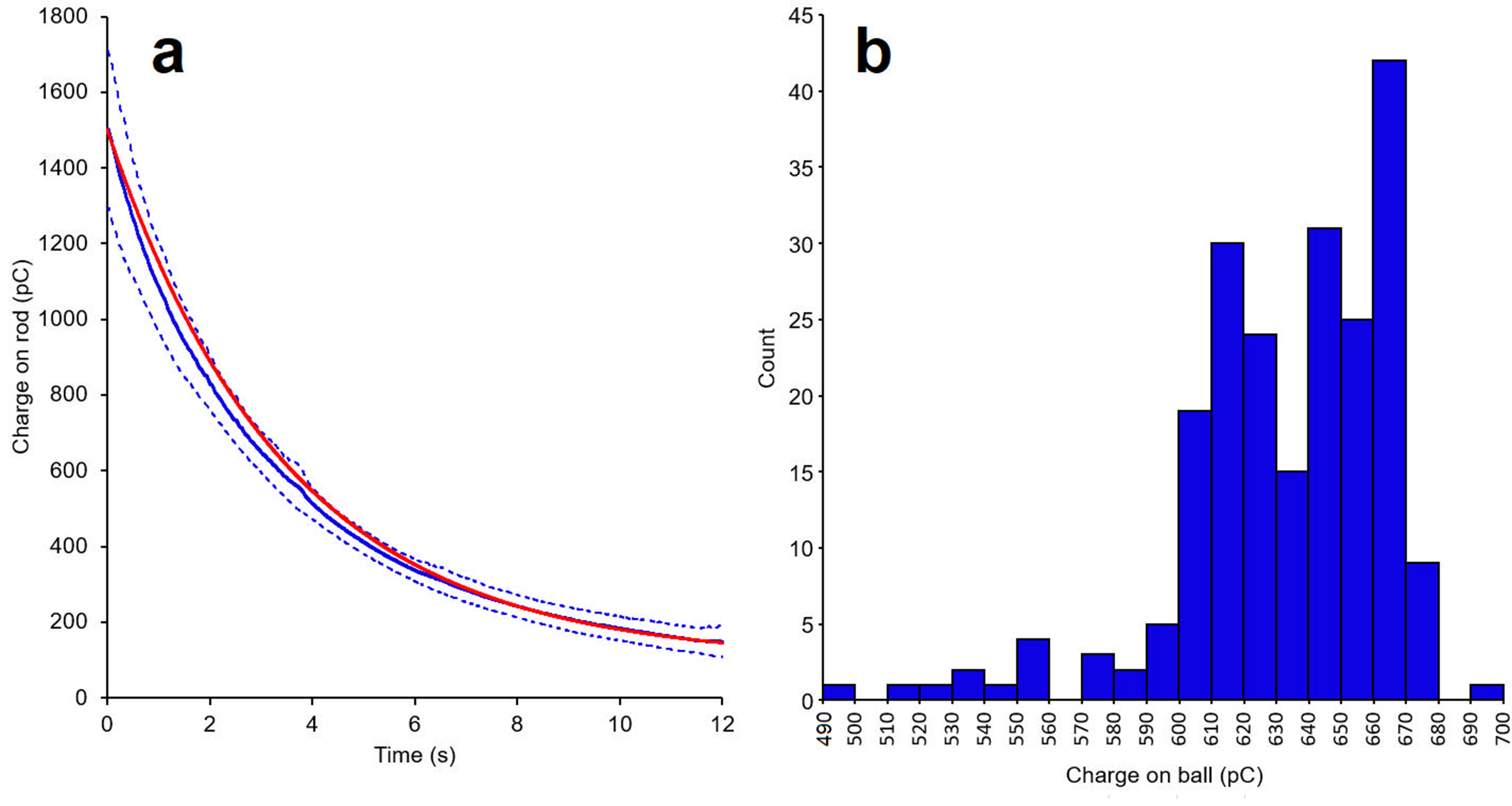

Supplement: Supplementary file 2 — a The mean charge decline on a triboelectrically charged nylon ball held in a Faraday pail (blue), dashed lines show SD. Red line indicates the modelled relationship used to calculate the charge on the ball at the point of touching the flower. b The modelled charges present on the nylon ball at the point of touching the flowers during the high charge experiments. (TIFF 292 kb) (PNG 30926 kb) [file 114_2021_1740_Fig5_ESM.png]

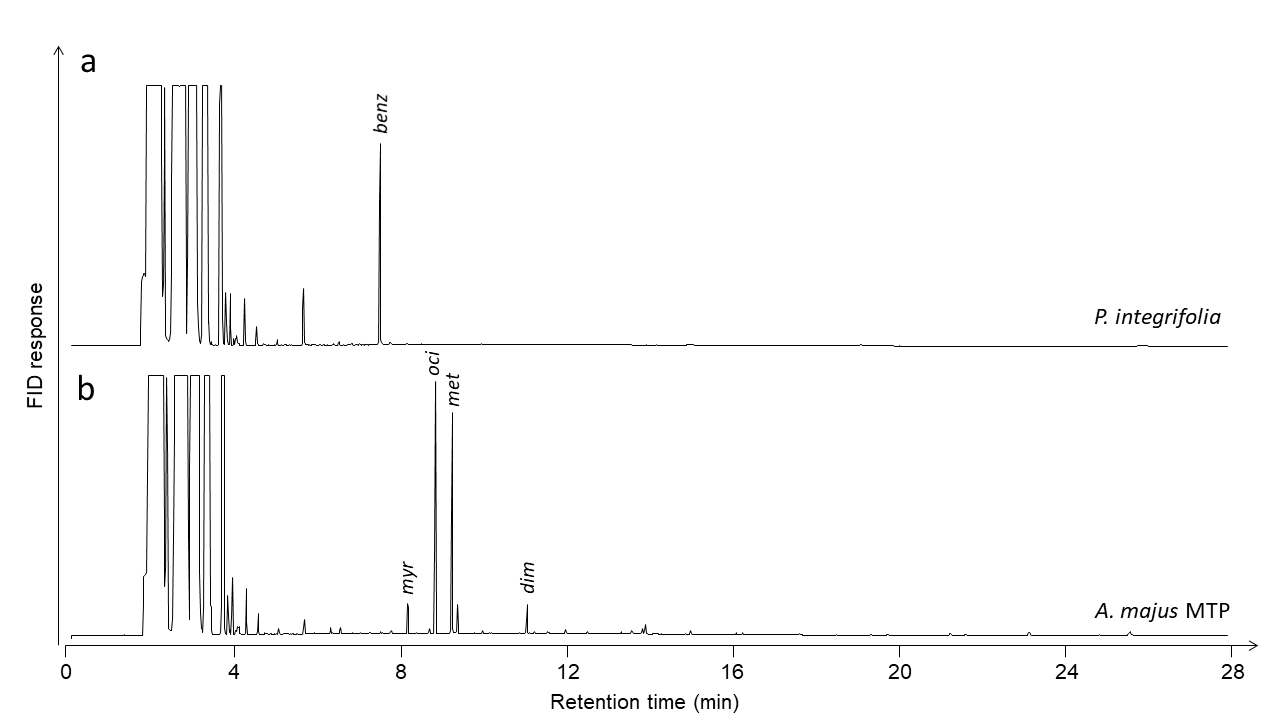

Supplement: Supplementary file 4 — The major compounds present in a P. integrifolia and b A. majus MTP. Peak labels indicate benzaldehyde (benz, KI=946), myrcene (myr, KI=990), (E)-ocimene (oci, KI=1043), methyl benzoate (met, KI=1064) and 3,5-dimethoxytoluene (dim, KI=1246). (JPEG 2654 kb) [file 114_2021_1740_MOESM3_ESM.jpeg]

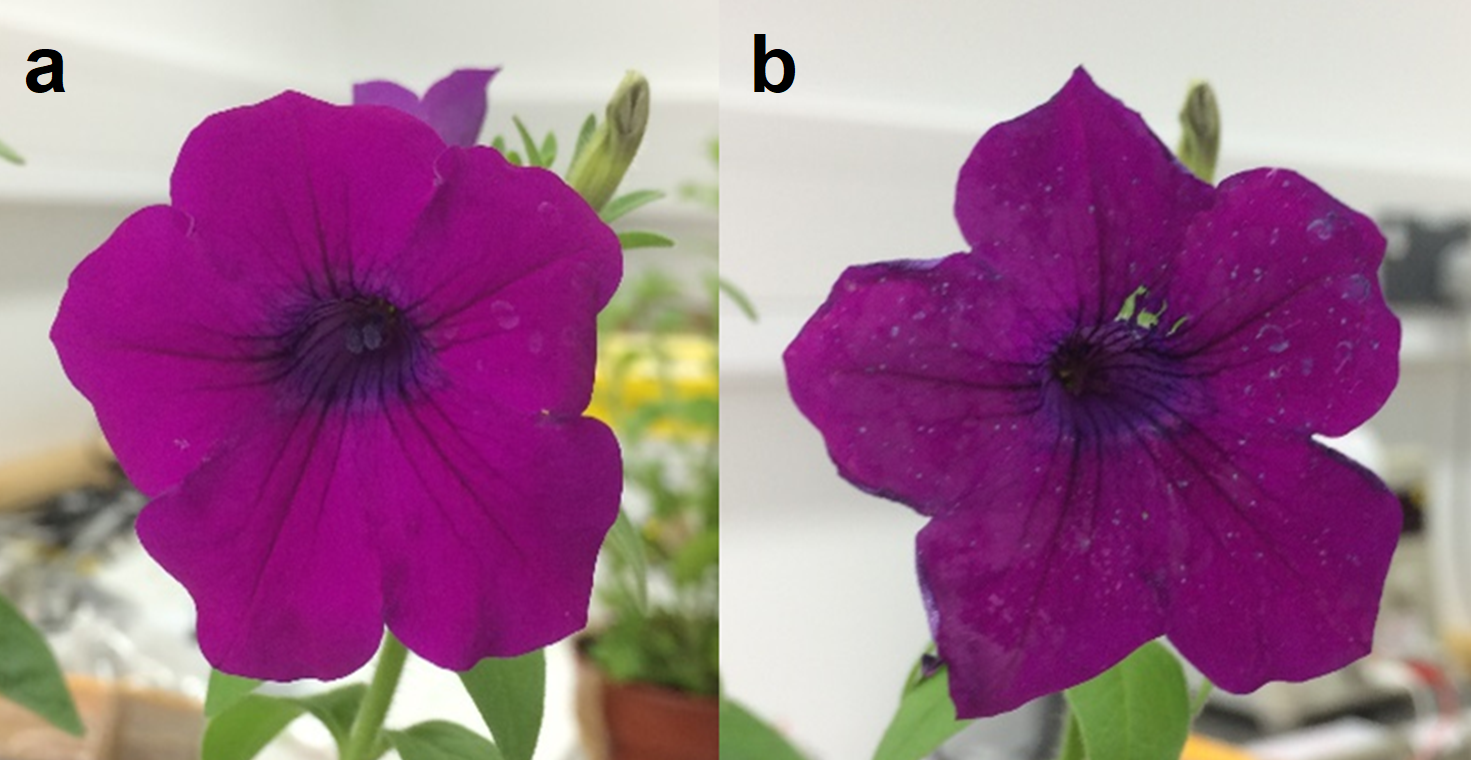

Supplement: Supplementary file 5 — The same Petunia integrifolia flower before (a) and after (b) a 2 h exposure to bumblebees showing mechanical wear and damage from bumblebee tarsi. (JPEG 1976 kb) [file 114_2021_1740_MOESM4_ESM.jpeg]
